# Galactic Cosmic Ray Particle Exposure Does Not Increase Protein Levels of Inflammation or Oxidative Stress Markers in Rat Microglial Cells In Vitro

**DOI:** 10.3390/ijms25115923

**Published:** 2024-05-29

**Authors:** Danielle S. Cahoon, Derek R. Fisher, Bernard M. Rabin, Stefania Lamon-Fava, Dayong Wu, Tong Zheng, Barbara Shukitt-Hale

**Affiliations:** 1 USDA-ARS, Human Nutrition Research Center on Aging at Tufts University, Boston, MA 02111, USA; danielle.cahoon@tufts.edu (D.S.C.); derek.fisher@usda.gov (D.R.F.); stefania.lamon-fava@tufts.edu (S.L.-F.); dayong.wu@tufts.edu (D.W.); tong.zheng@tufts.edu (T.Z.); 2 Department of Psychology, University of Maryland, Baltimore County (UMBC), Baltimore, MD 21250, USA; rabin@umbc.edu

**Keywords:** inflammation, oxidative stress, microglia, space-radiation, galactic cosmic rays

## Abstract

Astronauts on exploratory missions will be exposed to galactic cosmic rays (GCR), which can induce neuroinflammation and oxidative stress (OS) and may increase the risk of neurodegenerative disease. As key regulators of inflammation and OS in the CNS, microglial cells may be involved in GCR-induced deficits, and therefore could be a target for neuroprotection. This study assessed the effects of exposure to helium (^4^He) and iron (^56^Fe) particles on inflammation and OS in microglia in vitro, to establish a model for testing countermeasure efficacy. Rat microglia were exposed to a single dose of 20 cGy (300 MeV/n) ^4^He or 2 Gy ^56^Fe (600 MeV/n), while the control cells were not exposed (0 cGy). Immediately following irradiation, fresh media was applied to the cells, and biomarkers of inflammation (cyclooxygenase-2 [COX-2], nitric oxide synthase [iNOS], phosphorylated IκB-α [pIκB-α], tumor necrosis factor-α [TNFα], and nitrite [NO_2_^−^]) and OS (NADPH oxidase [NOX2]) were assessed 24 h later using standard immunochemical techniques. Results showed that radiation did not increase levels of NO_2_^−^ or protein levels of COX-2, iNOS, pIκB-α, TNFα, or NOX2 compared to non-irradiated control conditions in microglial cells (*p* > 0.05). Therefore, microglia in isolation may not be the primary cause of neuroinflammation and OS following exposures to helium or iron GCR particles.

## 1. Introduction

On exploratory missions, such as to Mars, astronauts will be exposed to galactic cosmic rays (GCR) which are composed of protons, alpha particles, helium (^4^He), and particles of high energy and charge (HZE particles), including iron (^56^Fe). Although HZE particles are less abundant than protons in the space environment, their high linear energy transfer (LET) makes them major contributors to the total dose and biological effects of exposures [1]. GCR particles are densely ionizing in contrast to other forms of ionizing radiation (IR, e.g., γ-rays and X-rays) [2], and exposure can induce neuroinflammation, oxidative stress (OS) [3], and cognitive deficits [2,3,4] at low doses. Furthermore, exposure to GCR particles may produce ‘accelerated aging’ in the central nervous system (CNS) [5] and increase risk for neurodegenerative disease [2]. However, the mechanisms underlying the progression of GCR-induced neurochemical and cognitive changes are unclear. Consequently, research on therapeutic strategies to attenuate the neurodegenerative effects that progress after radiation exposure is limited. Identifying cellular targets and mechanisms is necessary to advance the development of countermeasures against the neurodegenerative effects of GCR particle exposure.

Microglial cells, the resident macrophages of the central nervous system (CNS), are key regulators of neuroinflammation and OS in the CNS [6]. In response to a range of pathogenic stressors, microglia can change from a quiescent phenotype to an activated phenotype and produce cytotoxic mediators including pro-inflammatory cytokines (e.g., tumor necrosis factor-α [TNFα]), reactive oxygen species (ROS), and nitric oxide (NO) [7,8]. The excess production of these mediators can produce collateral damage to tissues beyond the site of the initial insult and promote further microglial activation [8,9]. Therefore, a vicious cycle of microglial activation and neurotoxicity can result, leading to chronic inflammation, OS, and neurodegeneration, long after the initial stressor dissipates.

Given that neuroinflammation and OS increase following exposure to the types of radiation encountered in space, microglial cells are a putative target in mediating GCR-induced neurochemical and cognitive deficits. However, research on microglial responses to GCR particles in vitro and in vivo is equivocal. While some animal studies using low to moderate dose (5 cGy–3 Gy) exposures to GCR particles (protons, ^4^He, ^56^Fe, ^16^O, ^48^Ti, and combined particles) have demonstrated microglial activation following irradiation [5,10,11,12,13], other studies did not find effects on microglial activation in vivo [14,15,16,17,18,19]. Additionally, to the best of our knowledge, microglial responses to GCR particles have not been assessed in vitro. Instead, in vitro research assessing the effects of radiation on microglia has used γ-rays and X-rays, which have different radiophysics and biological effects than heavier particles in GCRs due to different patterns of energy deposition [20,21,22]. Investigating microglial responses to GCR particles is needed to better understand and target radiation-induced neuroinflammation and OS following GCR exposures such as on exploratory missions beyond Earth’s magnetic field. As such, this study was designed as a proof-of-concept, preliminary study to establish an in vitro model for testing anti-inflammatory and antioxidant countermeasures against GCR-induced neuroinflammation and OS in microglia. This study assessed the effects of two GCR particles, ^4^He and ^56^Fe, on inflammation and OS in a microglial cell model (i.e., highly aggressively proliferating immortalized (HAPI) rat microglial cells) 24 h after exposure.

## 2. Results

### 2.1. Viability

Exposure to ^4^He or ^56^Fe did not produce changes in cell viability. Cell viability was on average 94.65 ± 0.93%.

### 2.2. Inflammation

**Figure 1:**
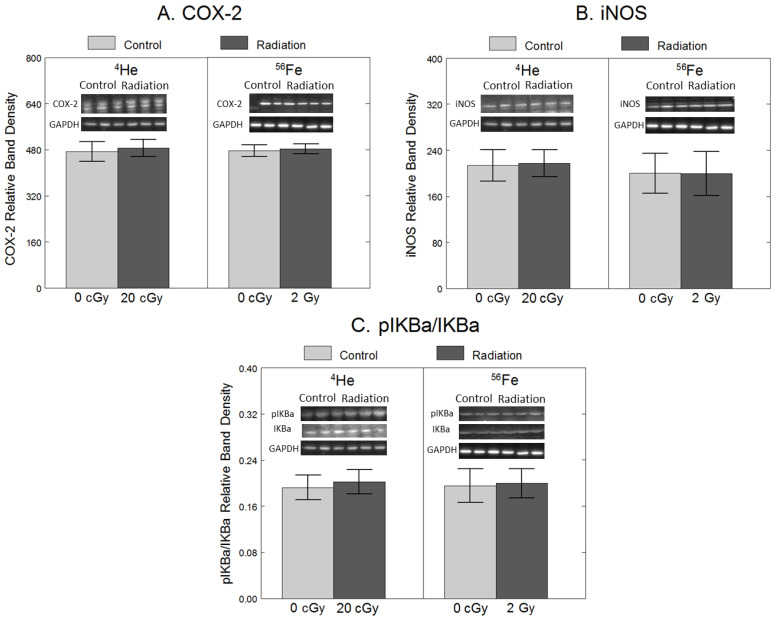
No significant effect of 20 cGy ^4^He or 2 Gy ^56^Fe on protein levels of pro-inflammatory enzymes in microglia. HAPI rat microglia were exposed to no radiation (0 cGy), 20 cGy ^4^He, or 2 Gy ^56^Fe. The following outcomes were assessed 24 h after irradiations using Western blot: (**A**) COX-2 protein levels, (**B**) iNOS protein levels, and (**C**) phosphorylated IκB-α protein levels, expressed as a ratio of total IκB-α. Data are presented as the mean ± SEM of six independent experiments (i.e., flasks) assessed in triplicate; example Western blot images of the markers and the loading control are shown at the top.

To assess the effects of ^4^He and ^56^Fe on inflammation, we quantified the protein levels of COX-2 and iNOS, intracellular enzymes responsible for producing pro-inflammatory prostaglandins [23] and nitric oxide (NO) [7]. Additionally, we quantified protein levels of pIκB-α, an indicator of increased activation of NF-κB, a transcription factor responsible for inducing the expression of inflammatory genes such as COX-2 and iNOS [23]. As shown in Figure 1, compared to non-irradiated cells, exposure to 20 cGy ^4^He did not have significant effects on COX-2 (F_1,34_ = 14, *p* = 0.71), iNOS (F_1,34_ = 0.00, *p* = 0.98) or pIκB-α/IκB-α (F_1,34_ = 0.84, *p* = 0.84), Similarly, 2 Gy ^56^Fe did not have significant effects on COX-2 (F_1,34_ = 0.09, *p* = 0.77), iNOS (F_1,34_ = 0.10, *p* = 0.75), or pIκB-α/IκB-α (F_1,34_ = 0.33, *p* = 0.57).

**Figure 2:**
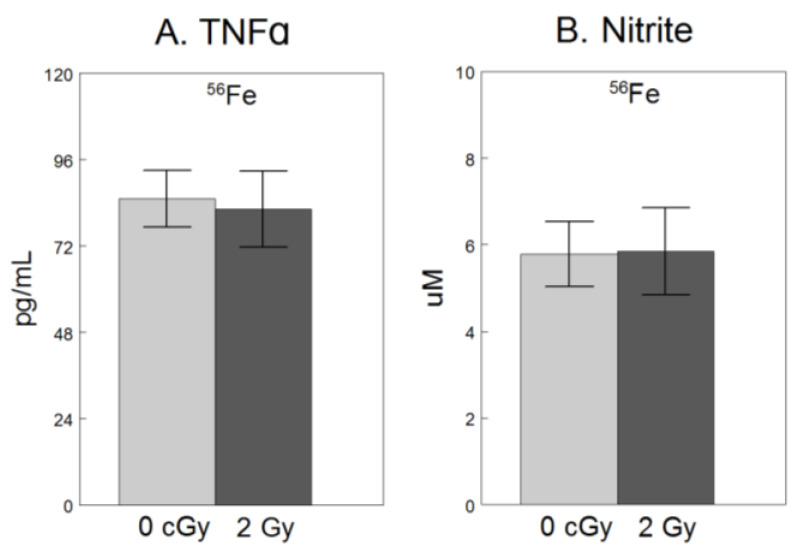
No significant effect of 2 Gy ^56^Fe on extracellular release of soluble pro-inflammatory factors in microglia. HAPI rat microglia were exposed to no radiation (0 cGy) or 2 Gy ^56^Fe. The following outcomes were assessed 24 h after irradiations in cell-conditioned supernatant (**A**) TNFα concentrations determined by ELISA, and (**B**) nitrite concentrations determined by Griess methods. Data are presented as the mean ± SEM of six independent experiments (i.e., flasks) assessed in triplicate.

For only the experiment using ^56^Fe, we assessed extracellular release of NO_2_^−^, a breakdown product of free radical NO and TNFα, a pro-inflammatory cytokine [9], both hallmarks of microglial activation [8,24]. As shown in Figure 2, compared to non-irradiated cells, exposure to 2 Gy ^56^Fe did not have significant effects on NO_2_^−^ (F_1,34_ = 0.18, *p* = 0.68), or TNFα (F_1,34_ = 0.07, *p* = 0.79).

### 2.3. Oxidative Stress

**Figure 3:**
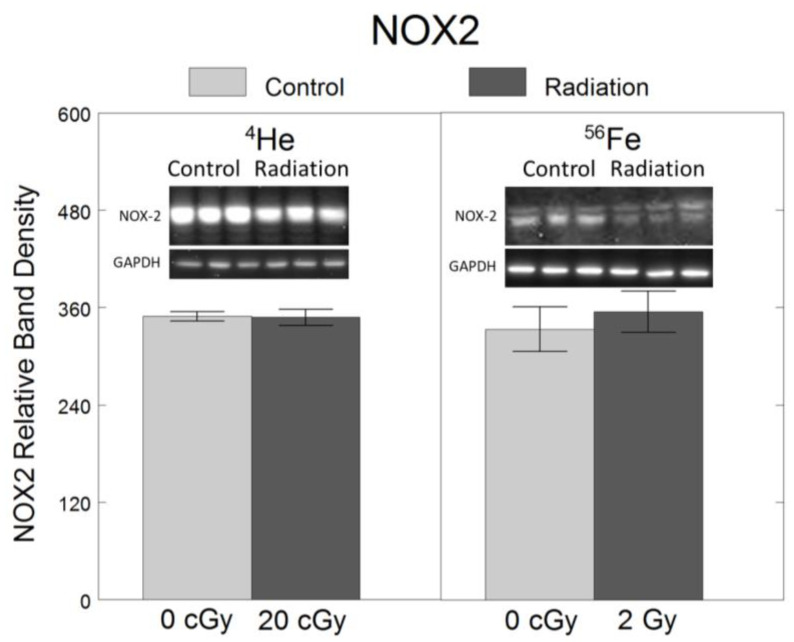
No significant effect of 20 cGy ^4^He or 2 Gy ^56^Fe on protein levels of oxidative stress-mediating enzyme NOX2 in microglia. HAPI rat microglia were exposed to no radiation (0 cGy), 20 cGy ^4^He, or 2 Gy ^56^Fe. NOX2 protein level was assessed 24 h following irradiation using Western blot. Data are presented as the mean ± SEM of six independent experiments (i.e., flasks) assessed in triplicate; example Western blot images of the markers and the loading control are shown at the top.

To assess the effects of ^4^He and ^56^Fe on oxidative stress, we quantified the protein levels of NOX2, a ROS-generating enzyme in microglia [6,7]. Relative to non-irradiated controls, no significant changes in NOX2 were observed following exposure to 20 cGy ^4^He (F_1,34_ = 0.005, *p* = 0.94) or 2 Gy ^56^Fe (F_1,34_ = 0.22, *p* = 0.64) (Figure 3).

## 3. Discussion

In this in vitro study, neither exposure to ^4^He (20 cGy) nor ^56^Fe (2 Gy) produced significant changes in the protein levels of inflammation (COX-2, iNOS, pIκB-α, TNFα, NO_2_^−^) or OS (NOX2) biomarkers in HAPI rat microglia compared to no irradiation. Results were similar for both particles. To our knowledge, this study is the first to assess the effects of exposure to GCR particles on inflammation and OS in microglia in vitro. Despite null findings, results contribute to a body of research examining the effects of space-like radiation on the CNS, and highlight the need to evaluate alternative mechanisms involved in the neuroinflammation and OS associated with particles in GCRs.

Previous work in rodents has shown that particles in GCRs, including ^4^He and ^56^Fe, can induce neuroinflammation, OS, and behavioral deficits [2,3,4]. However, the cellular mediators responsible for these changes have not been established. As key regulators of neuroinflammation and OS in the CNS [6], we hypothesized that microglia would play a pivotal role in the neurochemical and cognitive changes that result from radiation exposure. Several in vivo studies with rodents have demonstrated increased microglial activation indicated by expression of ionized calcium-binding adaptor molecule 1 (Iba-1) and/or CD68/ED-1 in brain tissue at acute and long-term durations following exposures to GCR particles [5,10,11,12,13]. Additionally, the depletion of microglia has been shown to limit irradiation-induced neurochemical and cognitive deficits [25]. However, other studies did not find effects of GCR particles at a range of post-exposure durations on microglial activation as measured by Iba and/or CD68/ED-1, despite increases in inflammation, OS, and/or other neurochemical changes [14,15,16,17].

Although in vivo studies provide important insight on integrated systemic responses, we assessed microglial responses to GCR particle radiation in vitro to isolate the putative role of this specific cell type in neurochemical changes. Previous work on microglial responses to ionizing radiation in vitro has been limited to high dose X-ray or γ-ray exposures. As with in vivo studies, findings on the effects of X-ray and γ-ray exposures on microglia activation, inflammation, and OS are inconsistent. For example, 10 Gy of ^137^Cs γ-ray has been shown to increase COX-2 protein levels and TNFα mRNA up to 24 h post-radiation, with or without changes in pIκB-α or NF-κB activity [26,27,28]. Another set of studies using ^137^Cs-137 γ-rays found increased COX-2 mRNA levels 24 h after higher doses (25 and 35 Gy) [29], but not with lower doses of 5, 15, or 16 Gy [29,30]. Irradiation with X-rays increased TNFα protein 24 h after 4 to 10 Gy, but not 2 Gy [31]. Another study found that 24 h after X-ray irradiation increased mRNA of COX-2 and TNFα at 2, 4, 6, 8, and 10 Gy, but did not produce any changes in iNOS or nitrite [32]

These mixed findings from in vitro studies using γ-ray and X-ray radiation in microglia highlight the gap in knowledge of microglial responses to radiation exposure. However, data from γ-ray and X-ray irradiation should not be extrapolated to predict responses to heavier particle radiation due to differences in energy distribution; γ-ray and X-rays are low linear energy transfer (LET) particles and deposit energy uniformly in cells and tissues, whereas higher LET particles, such as helium and HZE particles deposit energy along linear tracks in addition to scattering energy as delta rays outside the track [33]. Therefore, additional in vitro studies using high-LET particles in GCRs are necessary to understand the potential role of microglia in mediating responses to space-radiation.

Our findings suggest that microglia in isolation may be resistant to the effects of ^4^He and ^56^Fe exposures. However, because this was a proof-of-concept, preliminary study with the goal of establishing a model for countermeasure testing, results should be interpreted with caution. In this study, only protein levels of inflammation and OS biomarkers was assessed. Changes may occur in mRNA expression of these indicators, and/or at shorter or longer post-irradiation durations than at 24 h in this study. Furthermore, we assessed the effects of a single dose each for two particles in GCRs; higher doses may be necessary to cause microglial activation in vitro. Future studies should assess the effects of additional GCR particles, doses, combined particles, and post-irradiation time-points on inflammation and OS in microglia. Additionally, alternative indicators of microglial activation, such as morphology changes and secreted ROS, can provide further insight on microglial responses to radiation.

Acknowledging these limitations, and based on our consistent findings in an in vitro model, it is possible that microglia are not primarily responsible for irradiation-induced increases in neuroinflammation and OS with exposures to helium and iron GCR particles. Instead, these effects could be mediated by other cells in the CNS, such as astrocytes, or could be due to peripheral mechanisms, such as the activation of the vagus nerve or a compromised blood–brain barrier [33]. For example, studies have shown that exposure of just the body, but not the head, to GCR particles can produce neuroinflammation and OS in the brain, suggesting that peripheral mechanisms may mediate CNS effects [34,35]. Additionally, the response of microglia to GCR exposure may depend on the brain’s environment. In other words, microglia activation in response to radiation may require signals produced by other cells such as ROS, apoptosis signals, and damaged dendrites [33]. Therefore, future work should assess the effects of GCR particles on inflammation and OS in co-cultures of brain cells (e.g., neurons, astrocytes, and microglia).

## 4. Materials and Methods

### 4.1. Cell Culture and Irradiation Procedure

Highly aggressively proliferating immortalized (HAPI) rat microglial cells (generously provided by Dr Grace Sun, University of Missouri, Columbia, MO, USA) were maintained in Dulbecco’s modified Eagle’s medium (DMEM, Invitrogen, Grand Island, NY, USA) supplemented with 10% fetal bovine serum (FBS), 100 U/mL penicillin, and 100 μg/mL streptomycin at 37 °C in a humidified incubator under 5% CO_2_. Cells were transported by vehicle in T75 flasks from the Human Nutrition Research Center on Aging (HNRCA, Boston, MA, USA) to the NASA Space Radiation Laboratory (NSRL) at Brookhaven National Laboratory (BNL, Upton, NY, USA). Cells were then split into T25 flasks at a seeding density of 2.5 × 10^6^ cells/flask in DMEM without phenol red for 24 h prior to irradiation. During two separate runs, cells were exposed to a single dose of either 20 cGy (300 MeV/n) ^4^He or 2 Gy (600 MeV/n) ^56^Fe. Irradiations were performed at room temperature using a beam of ^4^He or ^56^Fe ions perpendicular to the plane of cells. Because the beam is horizontal, flasks were upended to a vertical position during irradiation. Dosimetry was provided by the NSRL staff using parallel plate ionizing chambers [36]. Doses were selected within the ranges of in vivo studies demonstrating increased microglial activation (up to 30 cGy ^4^He, up to 3 cGy ^56^Fe) [12,13], while minimizing the time required for flasks to be upended at room-temperature for irradiations.

Control cells for each run were not exposed to radiation (n = 6 flasks/group) but were treated similarly to those cells which were radiated (e.g., placed in a vertical position at room temperature), except they were not placed in the beam. Immediately following irradiation procedures, media was removed and cells were incubated in fresh media for 24 h. A 24 h post-irradiation interval was selected based on previous studies in microglia demonstrating increased inflammation (e.g., TNFα) 24 h after γ-ray or X-ray exposures [27,32,37,38]. After the 24 h post-irradiation period, cell lysates were prepared as described below, and transported back to the HNRCA by vehicle on ice. Samples were stored at −80 °C before processing for analysis of inflammation and OS biomarkers.

### 4.2. Cell Viability

Cell viability was assessed using the Trypan blue exclusion method [39]. In brief, microglial cells were seeded onto T25 flasks and experimental procedures were performed as above. Following the post-irradiation period (i.e., 24 h), cells were incubated with 500 μL trypsin/well for 5 min for detachment, and 50 μL of cell suspension was mixed with 50 μL of Trypan blue. Only dead cells with damaged membranes are permeable to Trypan blue and can be identified by their blue stain. The cell suspension was added to a hemocytometer and the number of blue-stained and non-stained cells were counted under a light microscope to calculate percentage of viable cells (non-stained/total cells × 100).

### 4.3. Western Blots

Cells were washed in ice-cold phosphate-buffered saline (PBS), resuspended and lysed by scraping in CelLytic-M Cell Lysis Reagent (Sigma-Aldrich, St. Louis, MO, USA) containing phenylmethylsulfonylfluoride (PMSF, 10 µg/mL), and centrifuged at 14,000 rpm for 10 min at 4 °C. The resultant supernatant lysate was used for blotting after total protein was quantified with the DC protein assay (BioRad, Hercules, CA, USA). Western blots were performed as described previously [40]. Primary antibodies were used at 1:1000 dilutions for inflammatory markers cyclooxygenase-2 (COX-2, Santa Cruz, Dallas, TX, USA), phospho- and total IκB-α (pIκB-α, IκB-α Santa Cruz, Dallas, TX, USA), and inducible nitric oxide synthase (iNOS, Millipore, Billerica, MA, USA), and oxidative stress marker NADPH oxidase (NOX2, Santa Cruz, Dallas, TX, USA), for incubation overnight at 4 °C. Glyceraldehyde 3-phosphate dehydrogenase (GAPDH, Santa Cruz, Dallas, TX, USA) was used as a protein-loading control marker. The signal was detected using an electrochemiluminescence (ECL) detection kit (BioRad, Hercules, CA, USA) and the optical density of antibody-specific bands was analyzed by the VisionWorks LS image acquisition and analysis software (version 8.1.2, UVP, Upland, CA, USA). Western blots were performed on six independent experiments per condition, assessed in triplicate. Values were normalized to GAPDH protein levels.

### 4.4. TNFα Enzyme-Linked Immunosorbent Assay (ELISA)

Secretion of inflammatory cytokine TNFα was quantified using ELISA according to manufacturer’s instructions (Invitrogen, Carlsbad, CA, USA). In brief, 50 µL of cell-conditioned supernatant or provided protein standards was added in duplicate to a 96-well plate and incubated for 2 h. Following washing, peroxidase-conjugated detection antibody was added to each well and incubated for 1 h. Wells were washed and substrate solution was added to each well for 15 min, followed by stop solution. Absorbance was read at 450 nm and the TNFα concentration in each sample was calculated from the linear equation derived from the standard curve of known concentrations of the cytokine. Values were normalized by total protein levels in cell lysates quantified with the DC protein assay (BioRad, Hercules, CA, USA).

### 4.5. Nitrite Quantification

To assess the production of free radical nitric oxide (NO) from HAPI cells, the extracellular release of nitrite (NO_2_^−^), a stable and nonvolatile breakdown product of NO, was measured in cell-conditioned supernatant by Greiss reagent kit (Invitrogen, Carlsbad, CA, USA) according to manufacturer’s instructions. In brief, 100 µL of supernatant from each flask was added in duplicate to a 96-well plate. Then, 25 µL of Griess reagent was added to each well and incubated at room temperature for 15 min. Absorbance was read at 548 nm and the concentration of NO_2_^−^ in each sample was calculated with the linear equation derived from the standard curve generated by known concentrations of NO_2_^−^. Values were normalized by total protein levels in cell lysates quantified with the DC protein assay (BioRad, Hercules, CA, USA).

### 4.6. Statistical Analysis

Statistical analyses were conducted in R (v3.8) using RStudio (v1.0.153), and results were considered statistically significant if the observed significance level was *p* < 0.05. One-way analysis of variance (ANOVA) was used to determine differences between irradiated and non-irradiated groups for each particle (^4^He or ^56^Fe) separately. Data are expressed as mean ± standard error of the mean (SEM).

## Data Availability

The data presented in this study are available on request from the corresponding author.
